# Elimination of the Sugar Transporter GAT1 Increased Xylanase I Production in *Trichoderma reesei*

**DOI:** 10.3389/fmicb.2022.810066

**Published:** 2022-01-26

**Authors:** Wenqiang Xu, Yu Fang, Mingyang Ding, Yajing Ren, Xiangfeng Meng, Guanjun Chen, Weixin Zhang, Weifeng Liu

**Affiliations:** State Key Laboratory of Microbial Technology, Shandong University, Qingdao, China

**Keywords:** *Trichoderma reesei*, xylanase, sugar transporter, GAT1, XYNI

## Abstract

The filamentous fungus *Trichoderma reesei* secretes large quantities of cellulases and hemicellulases that have found wide applications in industry. Compared with extensive studies on the mechanism controlling cellulase gene expression, less is known about the regulatory mechanism behind xylanase gene expression. Herein, several putative sugar transporter encoding genes that showed significant upregulation on xylan were identified in *T*. *reesei*. Deletion of one such gene, *gat1*, resulted in increased xylanase production but hardly affected cellulase induction. Further analyses demonstrated that deletion of *gat1* markedly increased XYNI production at the transcriptional level and only exerted a minor effect on XYNII synthesis. In contrast, overexpressing *gat1* caused a continuous decrease in *xyn1* expression. Deletion of *gat1* also affected the expression of *xyn1* and pectinase genes when *T*. *reesei* was cultivated with galacturonic acid as the sole carbon source. Transcriptome analyses of Δ*gat1* and its parental strain identified 255 differentially expressed genes that are enriched in categories of glycoside hydrolases, lipid metabolism, transporters, and transcriptional factors. The results thus implicate a repressive role of the sugar transporter GAT1 in *xyn1* expression and reveal that distinct regulatory mechanisms may exist in controlling the expression of different xylanase genes in *T*. *reesei*.

## Introduction

Lignocellulose including cellulose, hemicellulose, and pectin is the most abundant renewable carbon resource in nature (Rubin, 2008). Bioconversion of lignocellulose into fermentable sugars is of great significance regarding biofuel and bio-based chemical production for various industrial and synthetic applications (Himmel, 2007). In nature, a number of microorganisms are capable of efficiently degrading lignocellulose by secreting a large quantity of lignocellulolytic enzymes. Among others, the filamentous fungus *Trichoderma reesei* is one of the most prolific producers of cellulases and hemicellulases (mainly xylanases), which have found applications in many industrial fields for a long history (Bischof et al., 2016; Liu and Qu, 2021). Whereas *T*. *reesei* produces a much lower amount of xylanases compared to cellulases, these enzyme components play an indispensable role in increasing the accessibility of cellulases to cellulose that is embedded in hemicellulose matrix mainly composed of xylan, and therefore enhancing the overall efficiency of lignocellulose hydrolysis (Gupta et al., 2016). *Trichoderma reesei* secrets five endo-β-1,4-xylanases including XYNI–XYNV (Tenkanen et al., 1992, 2013; Torronen et al., 1992; Xu et al., 1998; Ogasawara et al., 2006; Herold et al., 2013) and two ß-xylosidases BXLI (Herrmann et al., 1997) and BXLII (Derntl et al., 2015) for efficient xylan degradation. XYNI and XYNII represent the major xylanases that are responsible for more than 90% of the total extracellular xylanolytic activity (Tenkanen et al., 1992; Torronen et al., 1992). Whereas the expression of XYNI and XYNII encoding genes (*xyn1* and *xyn2*) has been known to be readily triggered by xylan or Avicel, how the induction cascade is initiated is not clear yet. Understanding the underlying regulatory mechanism involved in xylanolytic gene expression would contribute to the knowledge-based strain design for increasing xylanase production.

During the past decades, extensive studies have been performed to investigate the molecular mechanism underlying the induced expression of cellulolytic and xylanolytic genes. Several transcription factors including XYR1 (Stricker et al., 2006; Furukawa et al., 2009; Derntl et al., 2013), ACE1 (Saloheimo et al., 2000; Rauscher et al., 2006), ACE2 (Aro et al., 2001; Würleitner et al., 2003; Mach-Aigner et al., 2010), ACE3 (Häkkinen et al., 2014; Zhang et al., 2019; Xue et al., 2020), and CRE1 (Strauss et al., 1995; Mach et al., 1996; Rauscher et al., 2006) have been shown to be involved in the regulation of most cellulase and xylanase genes (for details please refer to reviews Bischof et al., 2016; Shida et al., 2016). Whereas most transcription factors target the expression of both cellulase and xylanase genes, there are indications that distinct mechanisms involving specific regulatory factors are acting in the regulation of xylanase gene expression (Derntl et al., 2013). In this respect, two repressors, Xpp1 and SxlR, have been found to exert specific regulatory effects on the expression of xylanase genes but not cellulase genes. While Xpp1 fished out by the *xyn2* promoter in a pull-down assay acts as a repressor for both *xyn1* and *xyn2* (Derntl et al., 2015), SxlR plays a critical role in inhibiting the expression of GH11 xylanase genes including *xyn1*, *xyn2*, and *xyn5* (Liu et al., 2017).

The soluble mono- or oligo-saccharides released from lignocellulose enter the cytoplasm via membrane sugar transporters, acting as either nutrient molecules to provide energy and building blocks or as signaling molecules to regulate the biosynthesis of hydrolytic enzymes (Huberman et al., 2016). Therefore, sugar transporters have been considered to play important roles in coordinating nutrient acquisition and stringent control of enzyme production in the process of lignocellulose utilization. Whereas a number of *T*. *reesei* sugar transporters have been characterized regarding their *in vitro* activities in transporting sugars released from lignocellulose hydrolysis (Saloheimo et al., 2007; Ivanova et al., 2013; Zhang et al., 2013; Huang et al., 2015; Sloothaak et al., 2016; Nogueira et al., 2018; Havukainen et al., 2020, 2021a,b), relatively few of them have been found to be involved in the regulation of lignocellulolytic enzyme production. In particular, the cellobiose transporter Crt1 plays an essential role in activation of cellulase genes in *T*. *reesei*, regardless of the inducing carbon sources used (Ivanova et al., 2013; Zhang et al., 2013; Havukainen et al., 2020). Deletion of *crt1*, however, does not compromise but improves xylanase production (Zhang et al., 2013), implicating a difference in sugar transporter-involved induction mechanisms between cellulase and xylanase genes. On the other hand, the xylose transporter *Tr*STR1 has been demonstrated to play an important role in xylanase synthesis (Huang et al., 2015). Moreover, the sugar transporter Tr_69957 is not only involved in uptake of cellobiose, xylose, and mannose, but also participates in the expression regulation of both cellulase and xylanase genes in the presence of sugarcane bagasse (Nogueira et al., 2018). Given that *T*. *reesei* genome contains numerous genes encoding putative sugar transporters (Martinez et al., 2008), it is most likely that specific sugar transporters exist to be involved in regulating xylanase production. Identification of such transporters and unraveling the underlying regulatory mechanism would help to optimize the ratio of xylanase-to-cellulase in the *T*. *reesei* enzyme cocktail to improve the efficiency of lignocellulose saccharification.

In this study, several putative sugar transporter encoding genes that showed significant upregulated expression on xylan were identified in *T*. *reesei*. Deletion of *gat1*, which was previously identified to encode a galacturonic acid transporter, increased xylanase production on xylan with a predominant effect on *xyn1* expression. We also found that GAT1 is involved in the regulation of both pectinase and xylanase gene expression when *T*. *reesei* was cultivated with galacturonic acid as carbon source. Finally, transcriptome analyses of Δ*gat1* was performed to identify a gene set regulated by GAT1.

## Materials and Methods

### Strains, Media, and Culture Conditions

*Trichoderma reesei* QM9414 (ATCC 26921), a cellulase-enhanced derivative of the original strain QM6a, was used as the control strain throughout the study. QM9414–Δ*pyr4*, a strain created by deleting the uridine trophic marker gene *pyr4* in QM9414 (Wang et al., 2019), was used as the parental strain in this study. All *T*. *reesei* strains were maintained on malt extract agar. For transcription and enzyme production analyses, *T*. *reesei* cells were pre-cultured at 30°C for 36 h on a rotary shaker (200 rpm) in 1 L Erlenmeyer flasks containing 250 mL of Mandels-Andreotti (MA) medium (Mandels et al., 1962) and supplemented with 1% (v/v) glycerol. The mycelia were then harvested through filtration and washed twice with medium without any carbon source. Equal amounts of mycelia were transferred to fresh MA media containing 0.5% (w/v) beechwood xylan (Biosynth Carbosynth, United Kingdom), 1% (w/v) Avicel, 5 mM xylose, 0.5% (w/v) galacturonic acid, or 0.5% (w/v) beechwood xylan plus glucuronic acid (1 or 5 mM) as the sole carbon source, and were cultivated for the indicated time periods.

*Escherichia coli* DH5a was used for routine plasmid construction and amplification. Cells were cultured in lysogeny broth in a rotary shaker (200 rpm) at 37°C.

### Construction of Plasmids and *Trichoderma reesei* Mutant Strains

To delete *gat1* (Tr_*106330*), two DNA fragments corresponding to approximately 2.4 and 2.1 kb of *gat1* up- and downstream non-coding regions were amplified from QM9414 genomic DNA with the primer pairs F*106330*-up/R*106330*-up and F*106330*-down/R*106330*-down, respectively, and successively ligated into pDonor*pyr4* (Zhang et al., 2013) via BP-cloning (Invitrogen, United States). The resultant plasmid pDonor*106330pyr4* was used to transform *T*. *reesei* QM9414-Δ*pyr4* after linearization with I-*Sce*I. Deletion plasmids for *gat2* (Tr_*69026*), Tr_*82309*, and *xyn1* were similarly constructed. To construct the deletion plasmid for *gat2*, two DNA fragments corresponding to approximately 2.2 and 2.4 kb of *gat2* up- and downstream non-coding regions were amplified with the primer pairs F6*9026*up/R*69026*-up and F*69026*-down/R*69026*-down, respectively, and successively ligated into pDonor*pyr4* via BP-cloning to yield the disruption vector pDonor*69026pyr4*. For Tr_*82309*, approximately two 2.3 kb of up- and downstream fragments corresponding to Tr_*82309* non-coding regions were amplified with the primer pairs F*82309*-up/R*82309*-up and F*82309*-down/R*82309*-down, respectively, and successively ligated into pDonor*pyr4* via BP-cloning to create pDonor*82309pyr4*. For *xyn1*, approximately two 2.4 kb of up- and downstream of *xyn1* non-coding regions were amplified with the primer pairs F*xyn1*-up/R*xyn1*-up and F*xyn1*-down/R*xyn1*-down, respectively, and successively ligated into pDonor*pyr4* via BP-cloning to construct the pDonor*xyn1pyr4*. To delete *xyn2*, two DNA fragments corresponding to approximately 2.4 kb of *xyn2* up- and downstream non-coding regions were amplified with the primer pairs F*xyn2*-up/R*xyn2*-up and F*xyn2*-down/R*xyn2*-down, respectively. The upstream fragment was inserted into the *Hin*dIII/*Pme*I site of pUC19*-pyr4* (Lv et al., 2015) to generate pUC19*-pyr4-xyn2*up. The downstream fragment was then inserted into *Eco*RI-linearized pUC19*-pyr4-xyn2*up to obtain pUC19*-pyr4-xyn2*. The deletion plasmids including pDonor*69026pyr4*, pDonor*82309pyr4*, and pDonor*xyn1pyr4* were linearized with I-*Sce*I and the plasmid pUC19*-pyr4-xyn2* was linearized with *Ssp*I, followed by *T*. *reesei* transformation.

To complement the Δ*gat1* strain and detect the subcellular localization of GAT1, a 2.4 kb fragment of full-length *gat1* without stop codon was amplified from QM9414 genomic DNA with the primer pair Fre*106330*/Rre*106330*, and then inserted into the *Asc*I*/Pme*I site of pMDP*tcu*-*gfp*-T*trpC* (Lv et al., 2015) to yield the complementation plasmid wherein *gat1-gfp* was under the control of the *tcu1* promoter. All the primers used were listed in Supplementary Table 1.

Transformation of *T*. *reesei* was performed as previously described (Zhou et al., 2012). The transformants were selected on minimal medium for either uridine prototroph or for resistance to hygromycin (120 mg/mL). Anchored PCR was used to verify the correct integration events. Strains constructed in this study were listed in Table 1.

**TABLE 1:** Strains used in this study.

Strains	Description	References or source
*Trichoderma reesei* QM9414	A cellulase-enhanced derivative of *Trichoderma reesei* QM6a	ATCC 26921
QM9414-Δ*pyr4*	Deleting the uridine trophic marker gene *pyr4* in QM9414	Wang et al., 2019
Δ*gat1*	Deleting *gat1* (Tr_*106330*) in QM9414-Δ*pyr4*	This study
CpΔ*gat1*	Expressing *gat1*-*gfp* under the *tcu1* promoter in Δ*gat1*	This study
Δ*gat2*	Deleting *gat2* (Tr_*69026*) in QM9414-Δ*pyr4*	This study
ΔTr_*82309*	Deleting Tr_*82309* in QM9414-Δ*pyr4*	This study
Δ*xyn1*	Deleting *xyn1* (Tr_*74223*) in QM9414-Δ*pyr4*	This study
Δ*xyn2*	Deleting *xyn2* (Tr_*123818*) in QM9414-Δ*pyr4*	This study

### Growth Assays

To analyze the growth in liquid culture, *T*. *reesei* strains were pre-cultured in MA medium supplemented with 1% (v/v) glycerol at 30°C for 36 h. Mycelia were collected through filtration and washed twice with medium without any carbon source. Equal amounts of mycelia were then transferred to fresh MA medium containing 0.5% (w/v) beechwood xylan (Biosynth Carbosynth, United Kingdom) or 0.5% (w/v) galacturonic acid as the sole carbon source. After cultivation for the indicated periods, mycelia were filtered, dried at 80°C for 48 h, and finally weighed.

### Fluorescence Microscopy

To visualize GAT1-GFP, spores of CpΔ*gat1* were inoculated into minimal medium containing 1% (w/v) glucose. After 16 h-cultivation, the mycelia were subjected to fluorescence microscopy analyses. The fluorescence of GAT1-GFP was detected with a Nikon Eclipse 80i fluorescence microscope (Nikon, Melville, NY, United States), and the images were captured and processed by NIS-ELEMENTSAR software.

### Enzyme Activity and Protein Analyses

Cellobiohydrolase and β-glucosidase activities were determined by measuring the amount of released *p*-nitrophenol with *p*-nitrophenyl-D-cellobioside (*p*NPC; Sigma-Aldrich, United States) and *p*-nitrophenyl-β-D-glucopyranoside (*p*NPG; Sigma-Aldrich, United States) as substrates, respectively. Reaction was carried out in a 160 μL-reaction system containing 40 μL of diluted culture supernatant and 40 μL of substrate plus 40 μL of 50 mM sodium acetate buffer (pH 4.8). The mixture was incubated at 45°C for 30 min. For measurement of cellobiohydrolase activity, D-glucono-1,5-δ-lactone (1 mg/mL) was added to inhibit the activity of β-glucosidase (Deshpande et al., 1984). One unit (U) of *p*NPCase or *p*NPGase activity was defined as the amount of enzyme releasing 1 μmol of *p*NP per minute. For endoglucanase activity, measurement was carried out in a 120 μL-reaction mixture containing 60 μL of culture supernatant and 60 μL of 0.5% carboxymethylcellulose sodium salt (CMC, Sigma Aldrich, United States) dissolved in 50 mM sodium acetate buffer (pH 4.8) and was incubated at 50°C for 30 min. With glucose as standard, the release of reducing sugar in the mixture was determined using DNS method (Bailey et al., 1992). One unit of enzyme activity was defined as the amount of enzyme capable of releasing 1 μmol of glucose per minute. Xylanase activities were determined using beechwood xylan (Biosynth Carbosynth, United Kingdom) as substrate by measuring the amount of xylose released. Briefly, the assay was carried out in a 120 μL-reaction mixture containing 60 μL of diluted culture supernatant and 60 μL of 0.5% (w/v) xylan dissolved in 50 mM sodium acetate buffer (pH 4.8). The reaction mixture were incubated at 50°C for 10 min and the release of reducing sugar was determined using DNS method with xylose as standard. One unit of xylanase activity was defined as the amount of enzyme capable of releasing 1 μmol of xylose per minute.

SDS-PAGE and western blot were performed according to standard protocols. XYNI and XYNII were immunoblotted using polyclonal antibodies raised against peptides of XYNI (amino acids of 52–65) and XYNII (amino acids of 79–92), respectively.

### Quantitative Reverse Transcription PCR

Total RNA was extracted using TRIzol reagent (Sangong Biotech, China) and purified using the TURBO DNA-free kit (Ambion, United States) to remove gDNA according to the manufacturer’s instructions. Reverse transcription was performed using the PrimeScript RT reagent Kit (Takara Bio, Japan) according to the instructions. Quantitative PCR was performed on a Bio-Rad myIQ2 thermocycler (Bio-Rad, United States). Data was analyzed using the relative quantitation/comparative CT (ΔΔCT) method and was normalized to an endogenous control (*actin*). Three biological replicates were performed for each analysis and the results. Statistical analysis was performed using the student’s *t*-test analysis.

### Transcriptome Analysis

To analyze and compare the gene expression profiles of *T*. *reesei* on glucose and xylan, QM9414 was pre-cultivated in MA medium supplemented with 1% (v/v) glycerol for 36 h. An aliquot of mycelia was transferred to a fresh MA medium containing 1% (w/v) glucose or 0.5% (w/v) xylan, respectively, followed by continuous incubation at 30°C for 15 h. Similarly, to compare the gene expression profiles of Δ*gat1* and QM9414 on xylan, strains were pre-cultivated in MA medium supplemented with 1% (v/v) glycerol for 36 h and then transferred to a fresh MA medium containing 0.5% (w/v) xylan for further 15 h-cultivation. Filtered mycelia were quickly frozen in liquid nitrogen and stored at –80°C. RNA was extracted and RNA sequencing was performed at the Beijing Genome Institute (BGI; Shenzhen, China). Briefly, poly-(A) mRNA was purified and enriched from total RNA using Oligo-(dT) magnetic beads. Then random hexamer-primers were used to synthesize first-strand cDNA and second-strand cDNA by reverse transcriptase and DNA polymerase I. Double-stranded cDNA was then repaired to blunt ends, phosphorylated at the 5′ end and added the sticky end of “A” at the 3′ end. Suitable fragments were amplified by PCR using specific primers. The PCR products were thermally denatured into single strand and were circularized to obtain a single-stranded circular DNA library. Sequencing was performed by the BGISEQ-500 platform. Gene expression levels were calculated using FPKM (Fragments Per Kilobase of exon model per Million mapped fragments). Log_2_Ratio was the ratio of FPKM values for the treatment and control samples and indicates the degree of differential expression between two samples. The false discovery rate (FDR) was used to determine the *P*-value threshold in multiple testing. Differentially expressed genes were defined by default as those with FDR ≤ 0.001 and differences of more than two fold. Clean data was deposited in the NCBI Sequence Read Archive under the accession number of PRJNA771532 (QM9414 data on glucose/xylan) and PRJNA766839 (Δ*gat1*/QM9414 data on xylan).

### Sequence Analysis

Amino acid sequences from *T*. *reesei* were obtained from the NCBI^1^ or JGI^2^ databases.

## Results

### Identification of Putative Sugar Transporter Encoding Genes With Significant Induction on Xylan in *Trichoderma reesei*

In order to search for putative sugar transporters involved in the induced xylanase production, gene expression profiles of *T*. *reesei* QM9414 cells cultivated on glucose and xylan, respectively, were determined via transcriptome analyses. As expected, xylanolytic genes including *xyn1*, *xyn2*, *xyn4*, *xyn5*, and *bxl1* were all significantly induced on xylan compared to that on glucose (Supplementary Table 2). In addition, among the differentially expressed genes between glucose and xylan cultivation, eleven genes encoding putative sugar transporters displayed a significantly upregulated expression (Table 2). Given that the two top-ranked genes, Tr_*50894* and Tr_*104072*, have been reported to play roles in xylanase production or xylose transport (Saloheimo et al., 2007; Huang et al., 2015), we therefore chose the following three genes, Tr_*82309*, Tr_*106330*, and Tr_*69206*, to analyze their potential involvement in regulation of xylanase expression. While Tr_*106330* and Tr_*69206* have been recently demonstrated to encode sugar transporters (GAT1 and GAT2, respectively) capable of transporting galacturonic and glucuronic acids (Havukainen et al., 2021b), their roles in xylanase production is unclear.

**TABLE 2:** Putative sugar transporter encoding genes with significant induction on xylan in *Trichoderma reesei* QM9414.

Gene ID	QM9414_Glucose FPKM	QM9414_xylan FPKM	Log_2_(QM9414_xylan/QM9414_Glucose)	References
Tr_50894	0.41	745.12	10.82	Sloothaak et al., 2016 (*str1*)
Tr_104072	0.12	86.29	9.49	Huang et al., 2015; Havukainen et al., 2021a (*xlt1*)
Tr_82309	0.29	78.76	8.08	Havukainen et al., 2021a
Tr_69026	0.75	107.51	7.16	Havukainen et al., 2021b (*gat2*)
Tr_106330	5.68	450.89	6.31	Havukainen et al., 2021b (*gat1*)
Tr_53903	0.07	5.35	6.25	Unreported
Tr_22912	5.35	202.2	5.24	Saloheimo et al., 2007 (*hxt1*)
Tr_50618	1.62	59.53	5.19	Havukainen et al., 2021b (*frt1*)
Tr_62502	0.73	16.77	4.52	Unreported
Tr_3405	4.07	56.59	3.79	Havukainen et al., 2020; Ivanova et al., 2013; Zhang et al., 2013 (*crt1*)
Tr_68812	1.47	17.8	3.59	Unreported

### Disruption of *gat1* Improved Xylanase Production of *Trichoderma reesei* on Xylan

**FIGURE 1:**
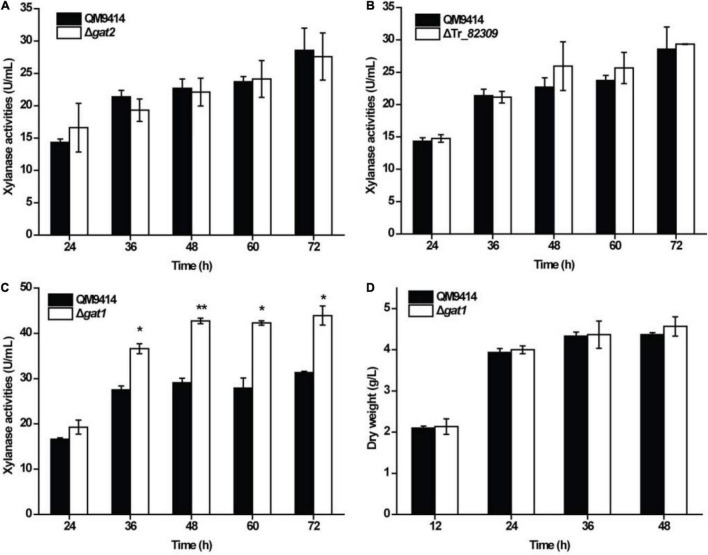
Deletion of *gat1* improved xylanase production of *Trichoderma reesei* on xylan. **(A–C)** Xylanase activity analysis of the culture supernatant from the QM9414 and mutant strains including Δ*gat2* **(A)**, ΔTr_*82309* **(B)**, and Δ*gat1* **(C)** cultivated on 0.5% (w/v) xylan for the indicated time periods. **(D)** Growth analyses of QM9414 and Δ*gat1* cultured on 0.5% (w/v) xylan. Values in this figure are the mean of three biological replicates. Error bars are the SD from these replicates. Significant differences (*t*-test *P < 0.05, **P < 0.01) were detected in the extracellular xylanase activities between QM9414 and Δ*gat1*. No significant differences were detected in the extracellular xylanase activities of Δ*gat2* or ΔTr_*82309* compared with that of QM9414. No significant differences were observed in biomass accumulation between Δ*gat1* and QM9414 when grown on xylan.

**FIGURE 2:**
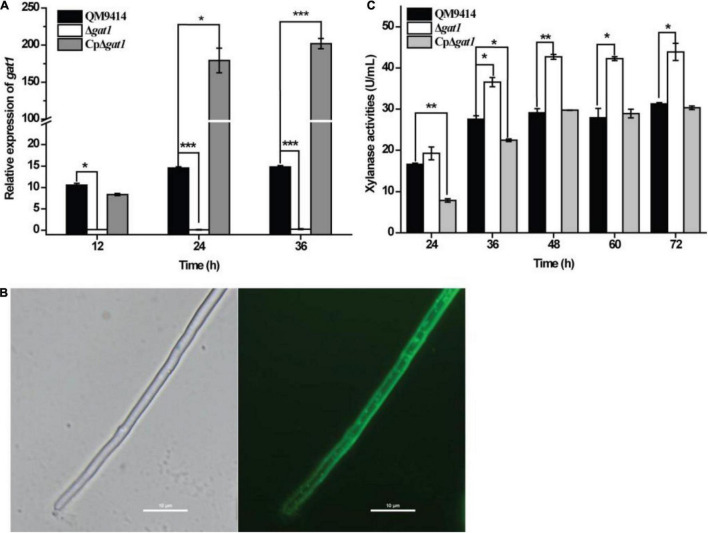
Complementation of Δ*gat1* with *gat1-gfp* overexpression reverted the elevated xylanase activity to the level of QM9414. **(A)** Quantitative RT-PCR analyses of the relative transcriptional levels of *gat1* in QM9414, Δ*gat1*, and CpΔ*gat1* cultivated on 0.5% xylan. **(B)** Fluorescence microscopic analyses of CpΔ*gat1* expressing *gat1*-*gfp* after cultivation on minimal medium containing 1% glucose for 16 h. **(C)** Xylanase activity analyses of the culture supernatant from QM9414, Δ*gat1* and CpΔ*gat1* strains cultivated on 0.5% xylan. Values in this figure are the mean of three biological replicates. Error bars are the SD from these replicates. Significant differences (*t*-test, *P < 0.05, **P < 0.01, and ***P < 0.0001) were detected in the transcriptional level of *gat1* and xylanase activities between QM9414 and Δ*gat1* or between QM9414 and CpΔ*gat1*.

To test the roles of Tr_*82309*, *gat1*, and *gat2* in xylanase production in *T*. *reesei*, they were individually deleted to result in the mutant strains ΔTr_*82309*, Δ*gat1*, and Δ*gat2*, respectively. Determination of extracellular xylanase activities demonstrated that, whereas disruption of *gat2* or Tr_*82309* had hardly any effect on xylanase production (Figures 1A,B), disruption of *gat1* resulted in a 38% increase in xylan hydrolytic activities compared to that of QM9414 strain (Figure 1C). Biomass accumulation analysis revealed little effect of *gat1* deletion on mycelial growth during the whole cultivation period (Figure 1D), indicating that the increment in xylanase production caused by *gat1* deletion did not result from differences in mycelial growth. Complementation of Δ*gat1* with an expression cassette of *gat1* fused to *gfp* revealed that the fluorescence signal was mainly dispersed at the periphery of the complemented mycelia (Figures 2A,B), indicating that GAT1 is appropriately located on the plasma membrane. Moreover, the restored expression of GAT1 fully reverted the elevated xylanase activity to the level of QM9414 (Figure 2C), supporting that the observed enhanced xylanase production was indeed caused by *gat1* deletion.

**FIGURE 3:**
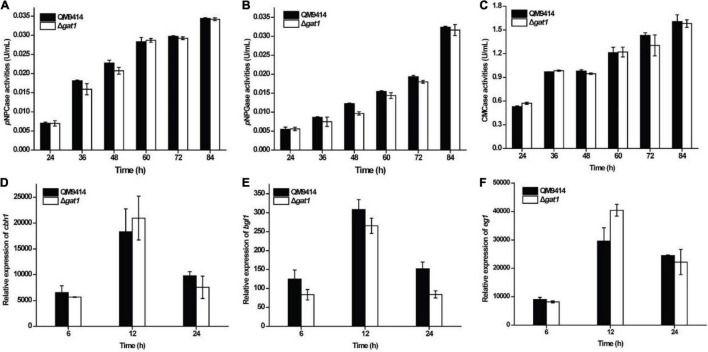
Deletion of *gat1* hardly affected cellulase production on Avicel cellulose. **(A–C)** Extracellular cellobiohydrolase **(A)**, ß-glucosidase **(B)**, and endoglucanase **(C)** activities of the culture supernatant from QM9414 and Δ*gat1*. **(D–F)** Transcriptional analyses of cellulase encoding genes including *cbh1* **(D)**, *bgl1* **(E)** and *eg1* **(F)** using quantitative RT-PCR. Strains were cultivated on 1% (w/v) Avicel cellulose for the indicated time periods. Values in this figure are the mean of three biological replicates. Error bars are the SD from these replicates. No significant differences were observed in cellulase activities or gene transcription between Δ*gat1* and QM9414.

To evaluate the role of GAT1 in cellulase production, Δ*gat1* was cultivated on Avicel cellulose and its performance was compared with that of QM9414. In contrast with the increased production of xylanase, Δ*gat1* showed quite similar cellulase production to that of QM9414 as determined by either extracellular cellulolytic activities or the relative transcriptional levels of the main cellulase genes (Figure 3).

### Deletion of *gat1* Predominantly Enhanced XYNI Production

**FIGURE 4:**
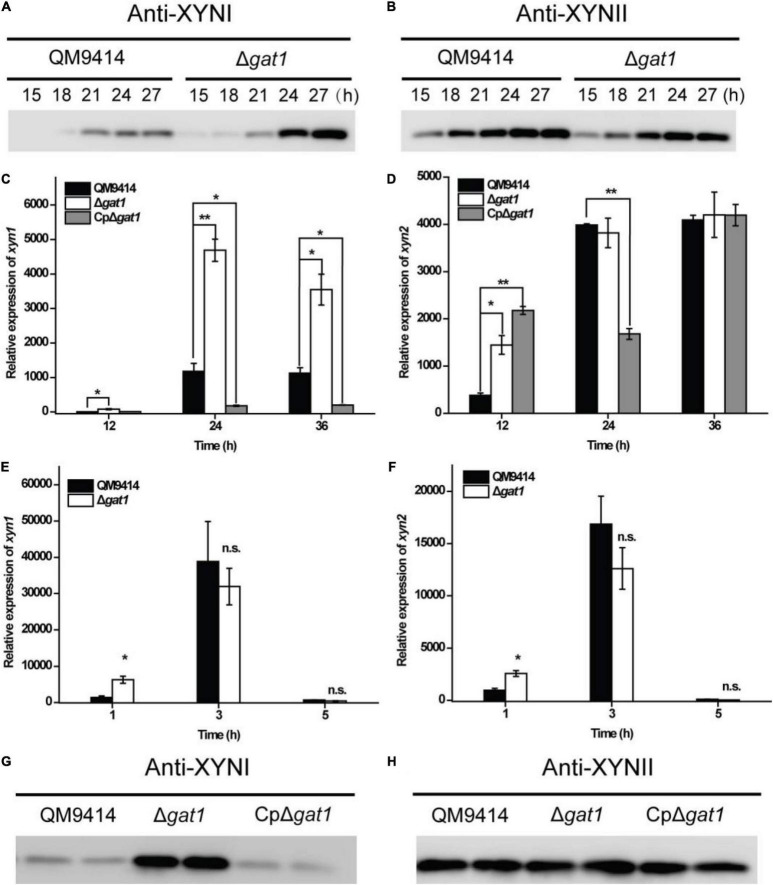
Deletion of *gat1* predominantly increased XYNI production but exerted a minor effect on XYNII synthesis. **(A,B)** Western blot analyses of XYNI **(A)** and XYNII **(B)** production in the culture supernatant of QM9414 and Δ*gat1* that were cultivated on 0.5% xylan for the indicated time periods. **(C,D)** Quantitative RT-PCR analyses of the relative transcriptional levels of *xyn1* **(C)** and *xyn2* **(D)** of QM9414, Δ*gat1* and CpΔ*gat1* cultivated on xylan. **(E,F)** Quantitative RT-PCR analyses of the relative transcriptional levels of *xyn1* **(E)** and *xyn2* **(F)** of QM9414 and Δ*gat1* cultivated on 0.5 mM xylose. **(G,H)** Western blot analyses of XYNI **(G)** and XYNII **(H)** production in the culture supernatant of QM9414, Δ*gat1*, and CpΔ*gat1* cultivated on xylan for 60 h. Values in this figure are the mean of three biological replicates. Error bars are the SD from these replicates. Significant differences (*t*-test, *P < 0.05, and **P < 0.01) were detected in the transcription of *xyn1* or *xyn2* between QM9414 and Δ*gat1*, or between QM9414 and CpΔ*gat1*. No significant difference (n.s.) was observed in the transcription level of *xyn1* or *xyn2* between QM9414 and Δ*gat1* after cultivation on 5 mM xylose for 3–5 h. For western blot analyses, equal amounts of culture supernatant were loaded for all strains. Three independent assays with quite similar results were performed and one representative figure is shown.

In order to further verify the promoting effect on xylanase production caused by *gat1* deletion, antibodies were raised against XYNI and XYNII, respectively, and western blot analyses were performed to detect the extracellular levels of these two main xylanases in *T*. *reesei*. To verify the specificity of antibodies, Δ*xyn1* and Δ*xyn2* were constructed in the background of QM9414–Δ*pyr4*, respectively. Western blot analyses verified that each antibody specifically picked out the corresponding xylanase and no cross-reactivity existed (Supplementary Figure 1). Further western blot analyses demonstrated that while the production level of XYNII in Δ*gat1* was quite similar to that of QM9414, XYNI production was significantly elevated in the absence of GAT1 (Figures 4A,B). Consistently, quantitative reverse transcription PCR (RT-PCR) analyses showed that disruption of *gat1* in QM9414 led to an up to 4-fold-enhancement in the expression of *xyn1* (Figure 4C). In contrast with *xyn1*, *gat1* deletion caused a moderate increase in *xyn2* expression only at the early growth phase (Figure 4D). Since xylose has been demonstrated to act as an inducer to trigger xylanase gene expression when present in lower concentrations (Mach-Aigner et al., 2010), the expression levels of *xyn1* and *xyn2* were analyzed in QM9414 and Δ*gat1* cultured with 0.5 mM xylose, respectively, and the results showed that their expression levels in Δ*gat1* were increased by 4.5- and 2.6-fold, respectively (Figures 4E,F), suggesting that deletion of *gat1* could also promote the expression of xylanase genes, especially *xyn1*, when in response to xylose.

It was also observed that overexpression of *gat1*–*gfp* in Δ*gat1*, to create the complemented strain, reversed the enhanced production of XYNI (Figure 4G). In consistence with reduced expression of *xyn1* (Figure 4C), the extracellular xylanase activity during the earlier growth stage was even markedly reduced in the complemented strain (Figure 2B). Again, the GAT1-GFP overexpression exerted only a minor effect on XYNII production (Figure 4H). Taken together, the above results demonstrated that *T*. *reesei* GAT1 is involved in the regulation of xylanase genes expression with *xyn1* as the major regulatory target.

### Deletion of *gat1* Affected Expression of Both Pectinase Genes and *xyn1* on Galacturonic Acid

**FIGURE 5:**
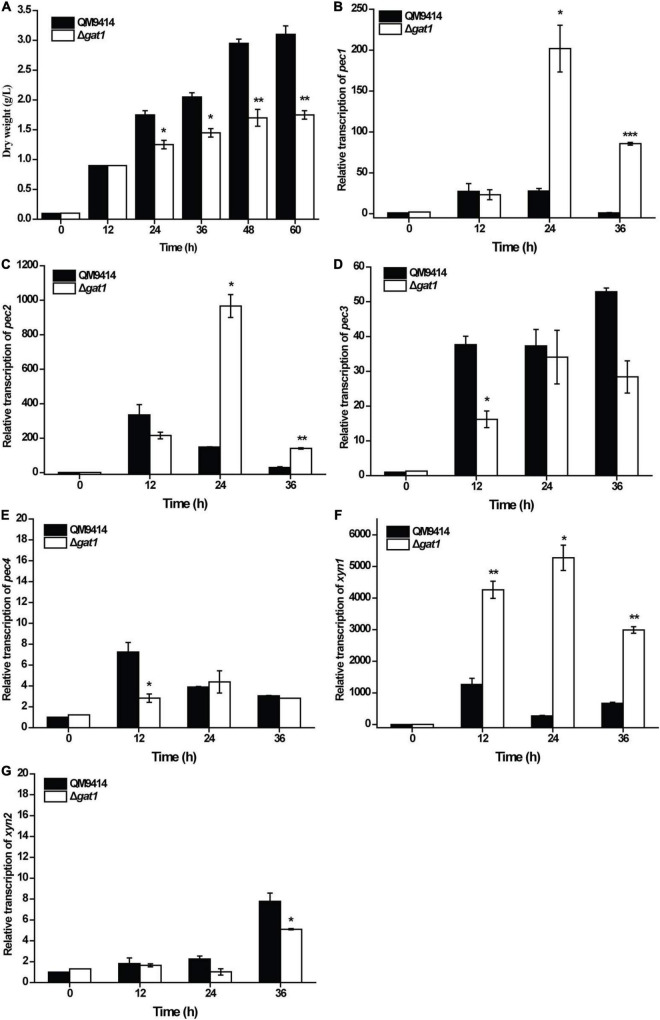
Deletion of *gat1* affected the expression of both pectinase genes xylanase genes upon galacturonic acid. **(A)** Growth analyses of QM9414 and Δ*gat1* with 0.5% (w/v) galacturonic acid as the sole carbon source. **(B–F)** Quantitative RT-PCR analyses of the relative transcriptional levels of *pec1* **(B)**, *pec2* **(C)**, *pec3* **(D)**, *pec4* **(E)**, *xyn1* **(F)** and *xyn2* **(G)** in QM9414 and Δ*gat1* cultivated with 0.5% (w/v) galacturonic acid as the sole carbon source. Values in this figure are the mean of three biological replicates. Error bars are the SD from these replicates. Significant differences (*t*-test, *P < 0.05, **P < 0.01, and ***P < 0.001) were detected in the biomass accumulation and relative transcription of the above genes between QM9414 and Δ*gat1*.

Given that GAT1 has been characterized as a galacturonic acid transporter (Havukainen et al., 2021b), we analyzed and compared the growth of Δ*gat1* and QM9414 using galacturonic acid as the sole carbon source. In consistence with the reported transporting activity of GAT1 toward galacturonic acid, Δ*gat1* displayed compromised biomass accumulation compared with QM9414 (Figure 5A), which was most probably caused by the impaired galacturonic acid transportation. Considering that galacturonic acid is a main hydrolysis product from pectin, the transcriptional levels of all four putative pectinase encoding genes, *pec1*, *pec2*, *pec3*, and *pec4* (Beier et al., 2020), were analyzed. Results showed that while all the four pectin hydrolytic genes can be readily induced by galacturonic acid in QM9414, disruption of *gat1* exerted differential effects on their transcriptional levels. Specifically, *gat1* deletion significantly increased the expression of *pec1* and *pec2*, but resulted in a reduced induction in the expression of *pec3* and *pec4* (Figures 5B–E). An induction of *xyn1* by galacturonic acid was also observed in QM9414 (Figure 5F). Interestingly, the absence of GAT1 caused a significantly enhanced transcription of *xyn1* compared to that of QM9414 (Figure 5F). By contrast, no increment in *xyn2* transcription was observed, but rather a moderate decrease was observed at 36 h of cultivation (Figure 5G). Together these data indicate that as a galacturonic acid transporter, GAT1 participates in the regulation of *xyn1* and pectinase gene expression when confronted with the hydrolytic products of pectin.

### Transcriptome Analysis of the Δ*gat1* Strain

**FIGURE 6:**
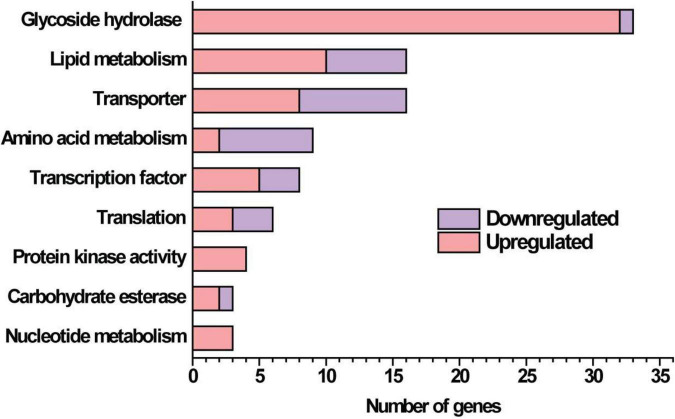
Functional category of differentially expressed genes in Δ*gat1* compared with QM9414. Transcriptome analysis identified a total of 255 differentially expressed genes between Δ*gat1* and QM9414. The 130 genes that encode hypothetical proteins and 27 unclassified proteins are not included in this figure.

Given that the absence of GAT1 enhanced xylanase production on xylan, we further undertook RNA sequencing (RNA-seq) to identify gene sets that may be regulated by GAT1 via profiling mRNA abundances in both QM9414 and Δ*gat1* strains after cultivation on xylan for 15 h. A Total of 255 genes were differentially expressed in Δ*gat1*, including 154 upregulated genes and 101 downregulated genes (Supplementary Database 1). Functional cluster analyses indicated that these differentially expressed genes are mainly enriched in the categories of glycoside hydrolases, lipid metabolism, transporters, amino acid metabolism, and transcription factors (Figure 6). In agreement with the above quantitative RT-PCR analyses, *xyn1* expression in the profile of Δ*gat1* was increased by ∼2.5-fold while no significant increase in *xyn2* expression was observed. The expression of *xyn5* was also increased by ∼2.5-fold. In addition, a number of genes encoding glycoside hydrolases involved in cellulose and hemicellulose degradation also showed significant upregulation in Δ*gat1* (Table 3), suggesting that GAT1 has a relatively extensive effect on expression of cellulase and hemicellulase genes in *T*. *reesei* under the cultivation condition of xylan.

**TABLE 3:** Glycoside hydrolase encoding genes with changed expression in Δ*gat1* on xylan.

Gene ID	Annotation	CAZy Family	QM9414_xylan FPKM	Δ *gat1*_xylan FPKM	Log_2_(Δ *gat1*_xylan/QM9414_xylan)
Tr_56996	Endo-1,4-β-mannosidase A	GH5	3.07	31.89	3.37
Tr_123232	Endoglucanase III	GH12	3.31	17.54	2.40
Tr_120312	Endoglucanase II	GH5	31.35	159.05	2.34
Tr_120961	Lytic cellulose monooxygenase	GH61	1.57	7.36	2.22
Tr_123283	α-L-Arabinofuranosidase	GH54	198.69	871.12	2.13
Tr_76672	β-Glucosidase I	GH3	35.5	141.77	1.99
Tr_122081	Endoglucanase I	GH7	35.54	136.8	1.94
Tr_72567	Cellobiohydrolase II	GH6	115.54	443.88	1.94
Tr_120873	Endo-1,3-α-glucosidase	GH71	93.62	355.7	1.92
Tr_59791	Chitinase	GH18	0.6	2.13	1.82
Tr_54242	Pectate lyase	GH15	5.79	20.43	1.81
Tr_69944	Maltase glucoamylase	GH31	44.89	157.7	1.81
Tr_123989	Cellobiohydrolase II	GH7	565.33	1985.98	1.81
Tr_112140	Putative polygalacturonase	GH28	20.03	64.77	1.69
Tr_120229	Xylanase III	GH10	1.94	6.25	1.68
Tr_69276	Putative glycoside hydrolase	GH30	10.17	32.63	1.68
Tr_81609	Glucoamylase	GH15	152.43	476.48	1.64
Tr_112392	Xylanase V	GH11	85.23	229.1	1.42
Tr_123726	Putative β-1,3-1,4-glucanase	GH16	2.21	5.77	1.38
Tr_27259	α-Galactosidase	GH27	3.07	7.92	1.36
Tr_49081	Xyloglucanase	GH74	53.78	137.45	1.35
Tr_74223	Xylanase I	GH11	3393.75	8519.31	1.32
Tr_76210	α-L-Arabinofuranosidase	GH62	42.8	107.3	1.32
Tr_62166	β-Mannosidase	GH2	16.94	41.9	1.30
Tr_70341	Chitosanase	GH75	28.34	68.9	1.28
Tr_110894	Galactan endo-1,6-β-galactosidase	GH5	50.08	119.68	1.25
Tr_73643	β-1,6-*N*-Acetylglucosaminyltransferase	GH61	1445.73	3381.06	1.22
Tr_76852	β-Glucuronidase	GH2	19.48	44.71	1.19
Tr_108671	β-Glucosidase	GH3	7.28	16.45	1.17
Tr_55319	α-L-Arabinofuranosidase	GH54	32.21	68.44	1.08
Tr_71394	Putative glycoside hydrolase	GH79	13.26	26.66	1.01
Tr_49976	Endoglucanase V	GH45	19.07	38.26	1.00
Tr_73248	Glucan 1,3-β-glucosidase	GH55	49.11	21.14	−1.21

Of the 16 putative transporter encoding genes with changed expression between Δ*gat1* and QM9414, four genes correspond to putative sugar transporters encoding genes (1 upregulated and 3 downregulated, Table 4). The gene encoding the sugar transporter Tr_69957 that is involved in cellobiose, mannose, and xylose absorption and cellulase and hemicellulase gene expression (Nogueira et al., 2018), showed an approximate 4.7-fold-upregulation. On the other hand, Tr_*62380* encoding the well-characterized glucose/xylose transporter STR3 (Sloothaak et al., 2016; Havukainen et al., 2021b) displayed a significant downregulation (∼11 fold) in Δ*gat1*, and the other two genes Tr_*76800* and Tr_*80875* encoding two putative sugar transporters with unknown functions were also downregulated. Moreover, eight transcriptional factor encoding genes were also differentially expressed in Δ*gat1* compared with QM9414 (Table 5). Tr_*122879*, the gene encoding the repressor Xpp1 (Derntl et al., 2015), was upregulated by ∼3-fold. Except for *xpp1*, none of the remaining seven genes has been described and their involvement in xylanase gene expression is unclear.

**TABLE 4:** Sugar transporter encoding genes with differential expression between Δ*gat1* and QM9414 on xylan.

Gene ID	QM9414_xylan FPKM	Δ *gat1*_xylan FPKM	Log_2_(Δ *gat1*_xylan/QM9414_xylan)	References
Tr_69957	33.73	160.38	2.24	Nogueira et al., 2018
Tr_76800	19.78	7.87	−1.32	Unreported
Tr_62380	12.67	1.13	−3.48	Sloothaak et al., 2016; Havukainen et al., 2021b (*str3*)
Tr_80875	8.44	3.69	−1.19	Unreported

**TABLE 5:** Transcription factor encoding genes with differential expression between Δ*gat1* and QM9414 on xylan.

Gene ID	QM9414_xylan FPKM	Δ *gat1*_xylan FPKM	Log_2_(Δ *gat1*_xylan/QM9414_xylan)	References
Tr_82512	8.44	78.02	3.20	Unreported
Tr_122879	150.28	443.04	1.55	Derntl et al., 2015 (*xpp1*)
Tr_112134	2.6	6.12	1.23	Unreported
Tr_75672	52.54	113.42	1.11	Unreported
Tr_60761	10.87	22.65	1.05	Unreported
Tr_47479	10.4	5.17	−1.01	Unreported
Tr_106009	36.71	16.96	−1.11	Unreported
Tr_59354	15.73	6.15	−1.35	Unreported

## Discussion

XYNI and XYNII, the two main extracellular xylanases of *T*. *reesei*, share quite a few structural and biochemical characteristics (Tenkanen et al., 1992; Torronen et al., 1992; Torronen and Rouvinen, 1995). Although expression of both *xyn1* and *xyn2* is highly induced on xylan and has been known to be co-regulated by several transcriptional factors including XYR1 (Stricker et al., 2006; Furukawa et al., 2009; Derntl et al., 2013; Xue et al., 2020), ACE3 (Häkkinen et al., 2014; Zhang et al., 2019; Xue et al., 2020), Xpp1 (Derntl et al., 2015), and SxlR (Liu et al., 2017), subtle differences have been noticed regarding their induction patterns and regulatory mechanisms. In particular, *xyn1* is virtually silenced in the presence of glucose while a partially constitutive expression of *xyn2* is allowed under the non-inducing carbon sources (Zeilinger et al., 1996). This phenomenon is consistent with the fact that *xyn1* is under direct control of the well-known carbon catabolite repressor CRE1 whereas *xyn2* is not (Mach et al., 1996). Moreover, the transcriptional repressor ACE1 only binds to the promoter of *xyn1* but not that of *xyn2* (Rauscher et al., 2006). To the contrary, the activator ACE2 does not exert apparent regulatory effect on *xyn1* expression while it contributes to the basal and induced expression of *xyn2* via binding to the xylanase activating element (XAE) in the *xyn2* promoter (Aro et al., 2001; Würleitner et al., 2003; Rauscher et al., 2006; Stricker et al., 2008). These published data implicate that different regulatory mechanisms work to fine tune the expression of *xyn1* and *xyn2*. In this respect, we found that a sugar transporter GAT1 exerts differential effects on the expression of *xyn1* and *xyn2* in response to either xylan or galacturonic acid. The absence of GAT1 caused a significant change in the expression of *xyn1* while *xyn2* expression was hardly affected. Given that the absence of another similarly functional galacturonic/glucuronic acid transporter GAT2 (Tr_69026) has no effect on xylanase production in *T*. *reesei* (Figure 1A), it is speculated that the role of GAT1 in regulation of xylanase expression is probably independent of its galacturonic/glucuronic acid-transporting activity. We also tested the effect of glucuronic acid, which might be released from beechwood xylan side chains into medium, on the production of xylanases upon xylan. Addition of glucuronic acid (1 or 5 mM) did not compromise but slightly promoted xylanase production in QM9414 (Supplementary Figure 2). These data excluded the possibility that the increment of XYNI production in Δ*gat1* resulted from the alleviated intracellular uptake of glucuronic acid which might repress xylanase gene expression.

Whereas previous study (Havukainen et al., 2021b) and our own assay (data not shown) showed that GAT1 does not have detectable xylose-transporting activity, its absence resulted in transcriptional changes of four putative sugar transporters encoding genes. Among others, the corresponding proteins of Tr_*69957* and Tr_*62380* have been previously demonstrated to play a role in xylose uptake (Sloothaak et al., 2016; Nogueira et al., 2018), which might consequently exert an effect on xylanase gene expression. Indeed, it has been reported that deletion of Tr_*69957* affects the expression of cellulase and xylanase genes in the presence of complex carbon source or simple sugars (Nogueira et al., 2018). Particularly, when cultivated with xylose as the sole carbon source, quite opposite changes in the transcription of *xyn1* and *xyn2* were observed in the Tr_*69957*-null mutant (Nogueira et al., 2018), implicating a different response pattern of xylanase gene expression to xylose. Although the upregulated expression of Tr_*69957* in Δ*gat1* may not cause a significant change in xylose uptake as shown by the similar growth of Δ*gat1* to QM9414 (Figure 1D), possibility exists that a subtle alteration in intracellular or extracellular xylose concentration together with the changed expression of the other sugar transporters, *e*.*g*., downregulated expression of Tr_*62380*, would consequently affected the expression of xylanase genes. On the other hand, it is also possible that deletion of *gat1* caused some variation in membrane sugar sensors yet to be known, which might not participate in direct sugar transportation, but act in the process of signal sensing and transduction from xylose or xylooligosaccharides molecules or their derivatives, and consequently affected xylanase gene expression.

Considering that transcriptional factors are well known to play direct regulatory roles in gene expression, we also identified changes in the expression of putative transcription factors encoding genes. While a total of eight relevant genes are differentially expressed between Δ*gat1* and QM9414, no significant changes in the transcriptional levels of genes encoding XYR1, ACE1, ACE2, or CRE1 were observed. Instead, the gene encoding the transcriptional repressor Xpp1 for both *xyn1* and *xyn2* was upregulated. Of note, previous reports showed that Xpp1 regulates the transcription of xylanase genes only at later cultivation stages (Derntl et al., 2015). In our results, deletion of *gat1* increased the transcription level of *xyn1* over the entire cultivation period (Figure 4C). Therefore, it was deemed that potential repressive effects brought by the upregulated expression of Xpp1 is probably overridden by other unknown factors. Specific transcriptional factors and the precise mechanism involved in this differential regulation of xylanase genes warrant further investigation.

GAT1 exhibits significant transporting activity toward galacturonic acid (Havukainen et al., 2021b), which is a main product from pectin hydrolysis. Given that xylan and pectin are both widely present in plant biomass, it is speculated that upregulated expression of GAT1 and also GAT2 on xylan may facilitate the simultaneous pectin utilization via galacturonic acid absorption. We found that galacturonic acid not only triggered expression of pectinase genes, but also that of *xyn1*. Deletion of *gat1* not only remarkably upregulated the expression of two major pectin hydrolytic genes, but also significantly increased the transcriptional level of *xyn1* on galacturonic acid. The precise mechanism remains unclear although it may involve as yet unknown mechanisms similar to that upon induction by xylan. One can surmise that a coordinated regulation of pectinase and xylanase genes were present in *T*. *reesei* to ensure efficient degradation of hemicellulosic biomass.

## Data Availability Statement

The original contributions presented in the study are included in the article/Supplementary Material, further inquiries can be directed to the corresponding author.

## Author Contributions

WL and WZ designed the project. WX, YF, MD, and YR performed the experiments. WZ and WX wrote the manuscript. WL revised the manuscript. All authors analyzed the data, read, and approved the final manuscript.

## Conflict of Interest

The authors declare that the research was conducted in the absence of any commercial or financial relationships that could be construed as a potential conflict of interest.

## Publisher’s Note

All claims expressed in this article are solely those of the authors and do not necessarily represent those of their affiliated organizations, or those of the publisher, the editors and the reviewers. Any product that may be evaluated in this article, or claim that may be made by its manufacturer, is not guaranteed or endorsed by the publisher.

## Footnotes

1
https://www.ncbi.nlm.nih.gov/

2
https://genome.jgi.doe.gov/
