# Supplementary material for: Elimination of the Sugar Transporter GAT1 Increased Xylanase I Production in Trichoderma reesei
Source: Front Microbiol. 2022 Jan 26;13:810066. doi: 10.3389/fmicb.2022.810066 (PMC8825865; doi:10.3389/fmicb.2022.810066)
Supplement: Supplementary file 2 [file Table_2.DOCX]

**Elimination of the sugar transporter GAT1 increased**

**xylanase І production in *Trichoderma reesei***

Wenqiang Xu, Yu Fang, Mingyang Ding, Yajing Ren, Xiangfeng Meng, Guanjun Chen, Weixin Zhang*, Weifeng Liu

State Key Laboratory of Microbial Technology, Shandong University, No.72 Binhai Road, Qingdao 266237, P. R. China

*Correspondence should be addressed to W Zhang. E-mail:zhangwx@sdu.edu.cn

**Supplemental Data**

**Table S1 Oligonucleotide primers used in this study.**

| **Primer** | **Application** | **Sequence (5'-3')** |
| --- | --- | --- |
| F*106330*-up | Primers used for deletion of Tr_*106330* | TAGGGATAACAGGGTAATACAACCTTATGCTGCTGAACCG |
| R*106330*-up | Primers used for deletion of Tr_*106330* | GGGGACAAGTTTGTACAAAAAAGCAGGCTAAGGTAGTGTTGAGCGTGGACTTGA |
| F*106330*-down | Primers used for deletion of Tr_*106330* | GGGGACCACTTTGTACAAGAAAGCTGGGTAGTAGCGTGCTCAGGCGGTTCT |
| R*106330*-down | Primers used for deletion of Tr_*106330* | ATTACCCTGTTATCCCTACGCTCGCTGTATTACTCGTCAA |
| F*69026*-up | Primers used for deletion of Tr_*69026* | TAGGGATAACAGGGTAATGTCAAGGGCAGCCAGGTTCTCG |
| R*69026*-up | Primers used for deletion of Tr_*69026* | GGGGACAAGTTTGTACAAAAAAGCAGGCTAAGGTTCGCCGGATGTGGATAGTG |
| F*69026*-down | Primers used for deletion of Tr_*69026* | GGGGACCACTTTGTACAAGAAAGCTGGGTACCTCCCACTTTTTCCCTTTCTC |
| R*69026*-down | Primers used for deletion of Tr_*69026* | ATTACCCTGTTATCCCTAAACCGCTATGTAGTTGCTTCAC |
| F*82309*-up | Primers used for deletion of Tr_*82309* | TAGGGATAACAGGGTAATTCAGCAAAGAAGGGAAGGACG |
| R*82309*-up | Primers used for deletion of Tr_*82309* | GGGGACAAGTTTGTACAAAAAAGCAGGCTAATGATGAGATGGTATCGCAAGAG |
| F*82309*-down | Primers used for deletion of Tr_*82309* | GGGGACCACTTTGTACAAGAAAGCTGGGTATCAAATCCCATCAGTCATCAGG |
| R*82309*-down | Primers used for deletion of Tr_*82309* | ATTACCCTGTTATCCCTAGCAGAAACACTCAGTCGCACC |
| F*xyn1*-up | Primers used for deletion of Tr_*74223* | TATGACCATGATTACGCCAAGCTTGGGCCCTGAATGATGGGAACAAGT |
| R*xyn1*-up | Primers used for deletion of Tr_*74223* | AGACACAGATCCGTTGACGTTTAAACATCAAACGGCATAGGAG |
| F*xyn1*-down | Primers used for deletion of Tr_*74223* | GTTTGGATGCAGTTGTCGACGGATCCGTATTGGGGTGGTTAGTG |
| R*xyn1*-down | Primers used for deletion of Tr_*74223* | TTGTAAAACGACGGCCAGTGAATTCAGTTCAGGAGTTCGCTTA |
| F*xyn2*-up | Primers used for deletion of Tr_*123818* | TAGGGATAACAGGGTAATGTAGTTAAGGGAACTCTTGTG |
| R*xyn2*-up | Primers used for deletion of Tr_*123818* | GGGGACAAGTTTGTACAAAAAAGCAGGCTAAGAAGGAAATGGAAAATCAA |
| F*xyn2*-down | Primers used for deletion of Tr_*123818* | GGGGACCACTTTGTACAAGAAAGCTGGGTACCATCAGGTCGGAAAGGTC |
| R*xyn2*-down | Primers used for deletion of Tr_*123818* | ATTACCCTGTTATCCCTAGCGGGGAAATGAAGGTGAC |
| Fre*106330* | Primers used for complementation of ΔTr_*106330* | GACCTGGTTGATACGACAATGGGCGTAACATCCAAGTTC |
| Rre*106330* | Primers used for complementation of ΔTr_*106330* | ATCTTGCAGGCCGGGCGAGACTTGATGTCTGCCCGCTC |
| qF*cbh1* | Primers used for quantitative RT-PCR | CTTGGCAACGAGTTCTCTT |
| qR*cbh1* | Primers used for quantitative RT-PCR | TGTTGGTGGGATACTTGCT |
| qF*eg1* | Primers used for quantitative RT-PCR | CGGCTACAAAAGCTACTACG |
| qR*eg1* | Primers used for quantitative RT-PCR | CTGGTACTTGCGGGTGAT |
| qF*bgl1* | Primers used for quantitative RT-PCR | AGTGACAGCTTCAGCGAG |
| qR*bgl1* | Primers used for quantitative RT-PCR | GGAGAGGCGTGAGTAGTTG |
| qF*xyn1* | Primers used for quantitative RT-PCR | AAACTACCAAACTGGCGG |
| qR*xyn1* | Primers used for quantitative RT-PCR | TTGATGGGAGCAGAAGATCC |
| qF*xyn2* | Primers used for quantitative RT-PCR | CGGCTACTTCTACTCGTACTG |
| qR*xyn2* | Primers used for quantitative RT-PCR | TTGATGACCTTGTTCTTGGTG |
| qF*actin* | Primers used for quantitative RT-PCR | TGAGAGCGGTGGTATCCACG |
| qR*actin* | Primers used for quantitative RT-PCR | GGTACCACCAGACATGACAATGTTG |
|  |  |  |

**Table S2 Xylanolytic gene expression in Δ*gat1* and QM9414 on xylan**

| **Xylanolytic gene** | **Protein ID** | **QM9414_Glucose FPKM** | **QM9414_xylan FPKM** | **Log_2_(QM9414_xylan/QM9414_Glucose)** |
| --- | --- | --- | --- | --- |
| *xyn1* | Tr_74223 | 0.01 | 1.85 | 7.53 |
| *xyn2* | Tr_123818 | 8.69 | 942.99 | 6.76 |
| *xyn4* | Tr_111849 | 0.46 | 89.47 | 7.60 |
| *xyn5* | Tr_112392 | 0.01 | 0.44 | 5.45 |
| *bxl1* | Tr_121127 | 0.83 | 872.88 | 10.03 |


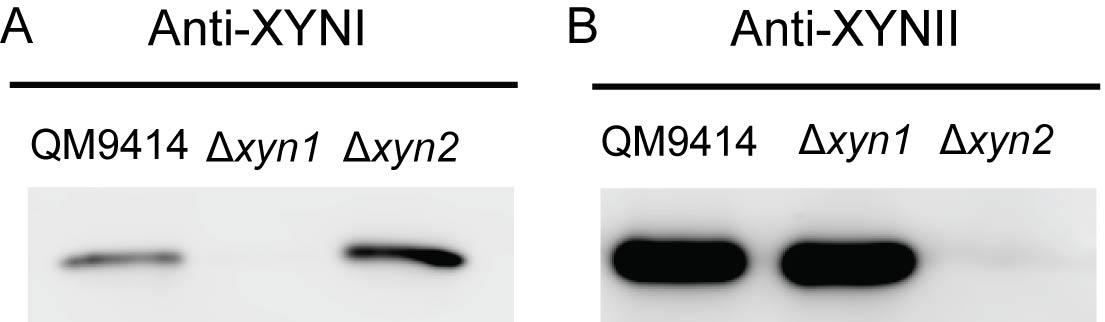


**Figure S1 Verification of specificity of antibodies against XYNІ and XYNⅡ.** Western blot analyses of extracellular supernatant of QM9414, Δ*xyn1* and Δ*xyn2* strains after induction on xylan for 60 h using antibodies against XYNІ (A) or XYNⅡ (B). Equal amounts of culture supernatant were loaded.


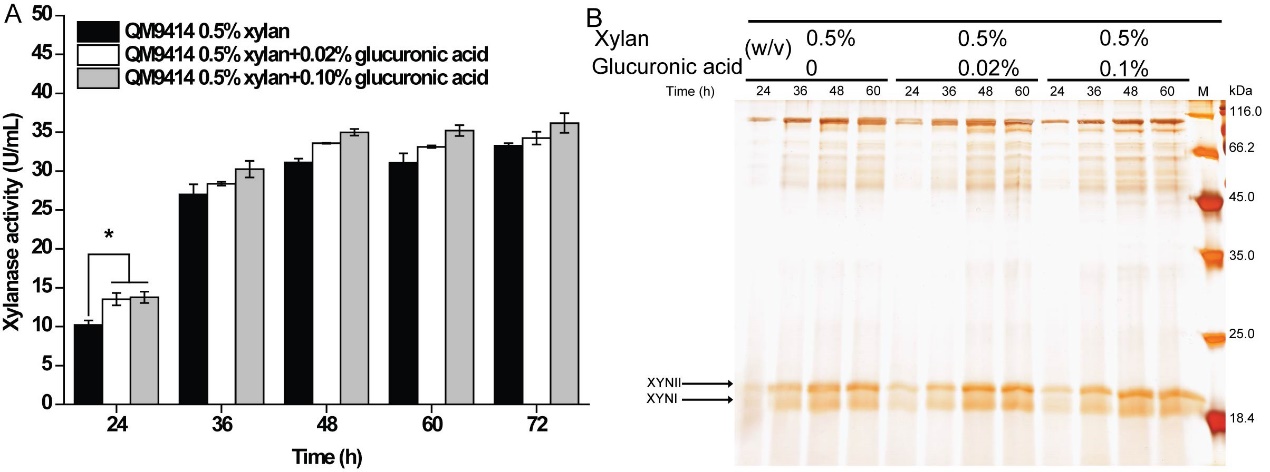


**Figure S2 The effect of addition of glucuronic acid on xylanase production of QM9414. (A)** Xylanase activities analysis of the culture supernatant from QM9414 cultivated in MA medium containing 0.5% (w/v) xylan and glucuronic acid (0.02% (1 mM) or 0.1% (5 mM) ). Values in the figure are the mean of three biological replicates. Error bars are the SD from these replicates. Significant differences (t-test *P < 0.05) were detected in xylanase activity at the early cultivation phase. **(B)** SDS-PAGE analysis of the culture supernatant of QM9414 cultivated in MA medium containing 0.5% (w/v) xylan and glucuronic acid (0.02% (1 mM) or 0.1% (5 mM) ).
